# miRNA Dysregulation in Cardiovascular Diseases: Current Opinion and Future Perspectives

**DOI:** 10.3390/ijms24065192

**Published:** 2023-03-08

**Authors:** Francesco Sessa, Monica Salerno, Massimiliano Esposito, Giuseppe Cocimano, Cristoforo Pomara

**Affiliations:** Department of Medical, Surgical and Advanced Technologies “G.F. Ingrassia”, University of Catania, 95121 Catania, Italy

**Keywords:** MicroRNAs (miRNAs), cardiovascular diseases (CVD), myocardial infarction, heart damage, heart failure, TheranoMIRNAs

## Abstract

MicroRNAs (miRNAs), small noncoding RNAs, are post-transcriptional gene regulators that can promote the degradation or decay of coding mRNAs, regulating protein synthesis. Many experimental studies have contributed to clarifying the functions of several miRNAs involved in regulatory processes at the cardiac level, playing a pivotal role in cardiovascular disease (CVD). This review aims to provide an up-to-date overview, with a focus on the past 5 years, of experimental studies on human samples to present a clear background of the latest advances to summarize the current knowledge and future perspectives. SCOPUS and Web of Science were searched using the following keywords: (miRNA or microRNA) AND (cardiovascular diseases); AND (myocardial infarction); AND (heart damage); AND (heart failure), including studies published from 1 January 2018 to 31 December 2022. After an accurate evaluation, 59 articles were included in the present systematic review. While it is clear that miRNAs are powerful gene regulators, all the underlying mechanisms remain unclear. The need for up-to-date data always justifies the enormous amount of scientific work to increasingly highlight their pathways. Given the importance of CVDs, miRNAs could be important both as diagnostic and therapeutic (theranostic) tools. In this context, the discovery of “TheranoMIRNAs” could be decisive in the near future. The definition of well-setout studies is necessary to provide further evidence in this challenging field.

## 1. Introduction

The function and development of the human heart are tightly regulated at multiple levels, starting from the differentiation from multipotent cells to embryonic cardiovascular progenitors, and then suppressing progenitor characteristics to allow differentiation and terminal maturation to arrive at aging processes [1]. MicroRNAs (miRNAs), which are small noncoding RNAs, are post-transcriptional gene regulators that can promote the degradation or decay of coding mRNAs, regulating protein synthesis [2]. In this way, these molecules are involved in various developmental, physiological, and pathological processes [3].

Over the past 15 years, a large number of experimental studies have contributed to clarifying the functions of several miRNAs involved in regulatory processes at the cardiac level [4,5]. Various studies have highlighted the pivotal role of certain miRNAs in hypertrophy, heart failure, and myocardial infarction, considering that they may be involved both in degenerative and reparative processes in cardiomyocytes [6,7,8,9,10,11]. Furthermore, given the intrinsic nature of the heart which is a muscle, several experimental studies focused on the involvement of the so-called myo-miRNAs as mediators and markers of cardiac damage [12,13]. Finally, other studies have shown that other miRNAs, not heart tissue or muscle-specific, may play a pivotal role in cardiovascular disease (CVD) [14,15,16,17,18].

In addition, therapeutic applications of miRNAs in the field of cardiovascular disease have been numerous in recent years, also considering that the death of cardiac cells in the aging heart cannot be replaced because of the arrest of cell division, so new therapeutic applications are needed to prevent cardiac damage. In recent years, attempts have been made to use miRNAs to control the cell cycle, thus trying to restore the regenerative potential of the mature myocardium [19,20,21].

In this context, this research field remains challenging for the international scientific community. This review aims to provide an up-to-date overview, with a focus on the past 5 years, of experimental studies in human samples to present a clear background of the latest advances in order to summarize our current knowledge and future directions.

## 2. Methods

A systematic review was conducted according to the PRISMA guidelines [22].

SCOPUS and Web of Science were used as the search engines, and searching was conducted from 1 January 2018 to 31 December 2022 to evaluate the present research on the association between miRNA dysregulation and cardiovascular diseases. The following keywords were used: (miRNA or microRNA) AND (cardiovascular diseases); AND (myocardial infarction); AND (heart damage); AND (heart failure).

### 2.1. Inclusion and Exclusion Criteria

The following exclusion criteria were used: (1) review, 531; (2) Book Chapter, 41; (3) Letter, 17; (4) Conference Paper, 8; (5) Editorial, 7; (6) Note, 5; (7) Retracted, 5; (8) Short Survey, 3; (9) Erratum, 2; articles not in English, 37.

The inclusion criteria were as follows: (1) Original Article, (2) Case Report, (3) Articles in English, (4) Human studies.

### 2.2. Quality Assessment and Data Extraction

F.S. and M.E. initially evaluated all the articles, evaluating the title, the abstract, and the whole text. M.S. then reanalyzed the articles chosen independently. In cases of conflicting opinions between the articles, they were submitted to C.P.

### 2.3. Characteristics of Eligible Studies

**Figure 1:**
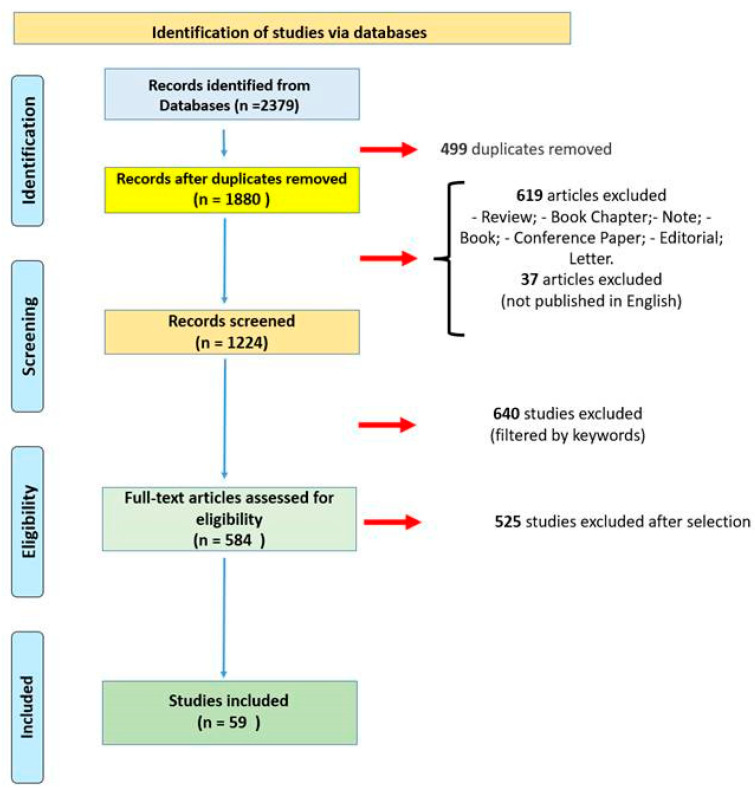
Flow diagram illustrating included and excluded studies in this systematic review.

A total of 2379 articles were collected. Of these, 499 duplicates were removed. A total of 619 articles did not meet the inclusion criteria and 37 articles were not in English. Of a total of 1224 eligible studies, through an electronic function of the SCOPUS database, 640 articles were excluded based on the following keywords: “Animal”; “Animals”; “Animal Experiment”; “Animal Model”; “Animal Tissue”; “Mouse”; “Animal Cell”; “Rat”; “Mice”; “In Vitro Study”; “Cell Proliferation”; “Rats”; “Disease Models, Animal”; “Mice, Inbred C57BL”; “Cells, Cultured”; “Sprague Dawley Rat”; “Rats, Sprague-Dawley”. In conclusion, 584 articles were further screened. After an accurate evaluation, 59 articles were included in the present systematic review (Figure 1).

## 3. Results and Discussion

**Figure 2:**
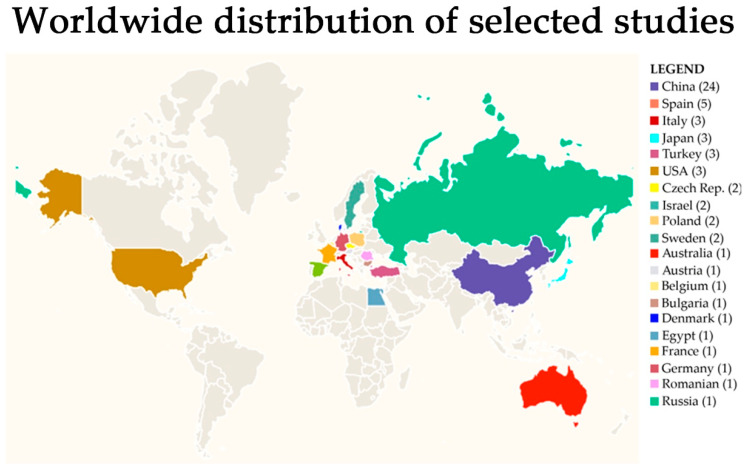
Distribution of selected studies by country.

Analyzing the nationality of the first author, as summarized in Figure 2, the research groups that contributed to the selected 59 articles were from China (24), Spain (5), Italy, Japan, Turkey, and the USA (3), Czech Republic, Israel, Poland, and Sweden (2), Australia, Austria, Belgium, Bulgaria, Denmark, Egypt, France, Germany, Romania, and Russia (1). Temporarily, the distribution of published articles by year of publication was 2018 (3), 2019 (6), 2020 (14), 2021 (22), and 2022 (14).

Focusing on the different miRNAs investigated, the roles of 223 miRNAs were tested. Among these, the miRNAs investigated in at least five studies were miR-133a-3p (10), miR-21, miR-499a-5p (8 times each), miR-1 (6), and miR-126 (5). This number refers only to those miRNAs that had been tested singularly, while a lot of studies used a screening approach based on microarray technologies in order to select the miRNAs to test in the second phase of their studies.

All selected articles are summarized in Table 1, following a chronological criterion.

### 3.1. miRNA Dysregulation and Heart Failure

The first study published in the included period was by Bayés-Genis et al. [23]; these authors investigated the role of 12 miRNAs, concluding that the levels of miR-1254 and miR-1306-5p were significantly associated with a risk of mortality and heart failure (HF) hospitalization. In this scenario, hypertrophic cardiomyopathy (HCM), a disease in which the heart muscle becomes thickened, is most often inherited and is the most common form of genetic heart disease. The main hallmarks of HCM are left ventricular hypertrophy, myocardial disarray, and interstitial fibrosis [82,83,84].

Wakabayashi et al. [43] demonstrated that different miRNAs could be involved in proinflammatory cytokine production, suggesting their involvement in ischemic heart disease (IHD). Moreover, the same authors demonstrated that the upregulation is significantly higher in Caucasians compared to Asian subjects, justifying the data that mortality from IHD is significantly lower in Japan than in other developed countries. Brundin et al. [46] reported that seven miRNAs were upregulated both in subjects suffering from idiopathic dilated cardiomyopathy (DCM) and IHD. Moreover, they described that miR-29-5p was significantly higher in DCM compared with IHD. Weldy et al. [45] reported that the high plasma levels of miR-28-3p, miR-433-3p, and miR-371b-3p are related to increased right ventricular size. Moscoso et al. [75] described the prognostic value of miR-499a and miR-125b in response to cardiac resynchronization therapy: the patients who positively responded to therapy showed lower plasma levels of these miRNAs. Eyyupkoca et al. [69] described that eight miRNAs (miR-23b-3p, miR-26b-5p, miR-199a-5p, miR-301a-3p, miR-374a-5p, miR-423-5p, miR-483-5p, and miR-652-3p) were associated with adverse left ventricular remodeling (ALVR), although only three, miR-301a-3p, miR-374a-5p, and miR-423-5p, in a significative manner. James et al. [71] performed a multiplex array profiling, demonstrating that miR-224-5p is more expressed in circulating vesicles of patients with reduced coronary flow reserve. Barbalata et al. [33] concluded that plasma levels of miR-142, miR-223, and miR-155 were higher in peripheral artery disease (PAD) patients with cardiovascular events (CVEs) compared with those without CVEs, while miR-92a is lower. Based on the results of this study, these miRNAs could be used as predictive factors of CVEs among PAD patients with good accuracy.

Tong et al. [63] demonstrated that miR-222 is more expressed in the first phase after MI. Moreover, these authors performed an in vitro study, demonstrating that MI damage could be attenuated by the inhibition of miR-222, considering that it may be involved in the regulation of the PI3K/AKT pathway during myocardial hypoxia-reoxygenation (HR) injury. Liu et al. [27] reported that different miRNAs were over-expressed in subjects with HF; in particular, they associated high plasma levels of miR-197-5p with adverse events after HF in patients under 50 years old. Garcia-Elias et al. [50] reported that plasma levels of miR-199a-5p and miR-22-5p are higher in patients with heart failure combined with reduced ejection fraction (HFrEF) and atrial fibrillation (AF), suggesting that they could be involved in arrhythmogenic mechanisms. Ben-Zvi et al. [34] demonstrated that various miRNAs may be involved in HF, showing upregulation of miR-125b-5p and miR-133-3p, and downregulation of miR-21-5p and miR-92b-3p. Masson et al. [25] highlighted a pivotal role for miR-132; in particular, miR-132 may be helpful to intensify strategies aimed at reducing re-hospitalization. Based on their results, higher levels of miR-132 were associated with more severe HF symptoms, suggesting that an anti-miR could be useful to reduce the risk for these patients. In the same year, Guo et al. [24] published a paper that highlighted the importance of miR-133a and miR-221 as potential HF diagnostic biomarkers, particularly when their levels were high in elderly patients. Zhang et al. [30] reported that patients with HF after MI had elevated miRNA-155 levels and poor cardiac function; for this reason, they hypothesized the use of this miRNA as a biomarker to assess the severity of the disease.

The identification of new molecular biomarkers in the early detection of HCM is a challenging objective in the research field. As reported by Thottakara et al. [62], the levels of miR-1, miR-3144, miR-4454, miR-495-3p, miR-499a-5p, and miR-627-3p were tested in the plasma samples of patients affected by HCM, and were higher than in healthy individuals. Interestingly, these authors described for the first time the correlation between high miR-4454 plasma levels and cardiac fibrosis in HCM, suggesting that miR-4454 could be used as a potential biomarker for cardiac fibrosis.

Nie et al. [38] reported that at the onset, miR-4763-3p and miR-4281 were upregulated in the plasma samples of patients suffering from fulminant myocarditis (FM), while their levels were restored as the clinical symptoms recovered. In this way, these miRNAs could be used as predictor factors of therapy-responsive patients.

### 3.2. miRNA Dysregulation and Acute Coronary Syndrome (ACS)

Acute coronary syndrome (ACS) is a term used to describe a series of conditions that are associated with a sudden reduction in blood flow to the heart. ACS includes three different pathological conditions: ST-segment elevation myocardial infarction (STEMI); non-ST-segment myocardial infarction (NSTEMI); and unstable angina (UA). A wide range of miRNAs have been investigated to clarify their role in ACS, considering that it is multifactorial and occurs in response to inflammation [85,86]. In this scenario, Mompeón et al. [74] reported that let-7g-5p, let-7e-5p, and miR-26a-5p plasma expression was inversely associated with serum levels of pro-inflammatory cytokines; this research group concluded that these biomarkers could be used as therapeutic/target biomarkers for ischemic heart disease.

Yu et al. [79] demonstrated that plasma levels of circulating miR-221 and miR-222 are upregulated in ACS patients, showing elevated levels which are positively associated with the severity of the coronary artery lesions. Elbaz et al. [35] concluded that seven miRNAs were significantly differentially expressed comparing ACS cases versus controls; in this way, miR-16, miR-92a, miR-122, miR-150, miR-186, miR-195, and miR-223-5p could be used as novel biomarkers for ACS. Shen et al. [59] investigated the role of 14 candidate miRNAs, demonstrating that the higher plasma levels of miR-4286 could be associated with an increased risk of ACS. Szelenberger et al. [61] analyzed miRNA expression starting from platelet samples in order to identify new molecular biomarkers of ACS. These authors analyzed the expression levels of 10 miRNAs, identifying 5 miRNAs overexpressed and 5 downregulated. Moreover, combining other clinical data, they concluded that in platelet samples, miR-142-3p could be used as a marker of modeling for ACS risk. Gager et al. [70] reported that high levels of miR-125b are related to a low survival rate after an ACS, suggesting that lower values could be related to improved long-term survival in patients with ACS and multivessel disease (MVD).

Coronary artery calcification (CAC) is an indicator of coronary artery disease (CAD), providing information about the cardiovascular risks of the patients involved in ACS. CAC is frequently associated with different chronic inflammatory diseases, such as diabetes, chronic kidney disease, and aging, that are widely diffused in developed countries; for this reason, the identification of theranostic markers could be very useful. Yang et al. [78] reported that the under-regulation of miR-29 in serum samples may be considered an important signal of early identification of CAC. In agreement with this study, He et al. [52] concluded that lower plasma levels of miRNA-29b may be used as independent risk factors for the incidence of cardiovascular events (CVEs) and CAC. Lu et al. [54] focused their study on the role of miR-27b: high levels of this miRNA may predict the occurrence of asymptomatic carotid artery stenosis (ACAS), with a high risk of generating subsequent cerebral ischemia events (CIEs).

Zhelankin et al. [67] reported that an increase in miR-146a-5p and miR-21-5p represents an indicator of ACS; at the same time, a downregulation of miR-17-5p could be considered a general biomarker of CAD. Zhou et al. [81] concluded that the circulating level of miR-133a was significantly higher in patients with periprocedural myocardial injury before and after the procedure, concluding that this biomarker may be used for early identification of stable CAD patients. Coban et al. [48] reported miR-18a-3p and miR-130b-5p as biomarkers of CAD, indicating its development and progression. The role of miR-150-5p, miR-29a-3p, and miR-30a-5 has been investigated by Silverman et al. [40] who highlighted the high plasma levels of these miRNAs as being related to SCD in subjects suffering from CAD. These results are confirmed by Yamada et al. [65] who reported that levels of miR-21 and miR-29a are higher in subjects suffering from CVD, while a low level of miR-126 seems to be related to an increased risk of death. Zhu et al. [31] summarized that serum levels of miR-182-5p and miR-5187-5p could be used as diagnostic biomarkers for CVD, providing diagnostic information for discriminating unprotected left main coronary artery disease patients from non-coronary artery disease. Asulin et al. [32] reported that the expression values of different miRNAs could be related to the etiology of rheumatic from idiopathic valvulopathies, improving our knowledge of the mechanisms underlining aortic valve (AV) disease. Sacchetto et al. [58] performed a case-control study, concluding that the upregulation in the plasma sample of miR-185-5p could be used as a promising biomarker in arrhythmogenic right ventricular cardiomyopathy (ARVC) patients.

Liu et al. [37] reported that different miRNAs are upregulated both in stable and unstable angina (UA) compared with subjects with normal coronary arteries; in particular, they suggest that these circulating miRNAs could be used as potential biomarkers of UA. Ling et al. [36] reported that miR-21 (less expressed compared with controls) and miR-126 (more expressed compared to controls) could be useful markers for predicting AMI and UA. Elgebaly et al. [49] reported that the expression levels of miR-137 and miR-106b-5p could be used as reliable biomarkers for UA and acute STEMI patients. Turky et al. [42] confirmed the importance of testing miR-133 in the plasma of subjects suffering from CVD. Particularly, this miRNA was higher in NSTEMI than UA patients; moreover, a significant upregulation is reported in the STEMI group compared to the NSTEMI group. Xiao et al. [64] concluded that miR-146a could be used as a new biomarker for adverse prognosis of STEMI; in particular, it may exert its function and its pathogenesis by targeting S100A12, a protein that is involved in the inflammatory response. J. Li et al. [72] analyzed the predictive value of serum miR-203 for acute STEMI, concluding that this miRNA represents a potential new biomarker in the early prediction of STEMI. Finally, Li et al. [26] demonstrated that the plasma levels of miR-208, miR-494, miR-499, and miR-1303 were higher in the tested group compared to controls; at the same time, the authors concluded that their predictive value is not superior to high-sensitivity cardiac troponin T (hs-cTnT) as myocardial markers in the diagnosis of early acute myocardial infarction (MI).

Neiburga et al. [57] examined the role of 17 miRNAs, identifying different risk factors: in particular, miR-629-5p and miR-98-5p were indicated as risk factors for acute MI. Gevaert et al. [51] investigated the role of miR-181c in response to exercise training in patients after MI: the circulating levels of this miRNA could be useful to identify low responders (LR) prior to training, giving the opportunity to plan a specific recovery activity. Su et al. [41] reported that circulating miR-1 within 3 h of acute chest pain could be of potential diagnostic value for AMI and could be classified as an independent risk factor for the prognosis of AMI. Hromadka et al. [53] identified two miRNAs that could be considered predictor markers for thrombotic events; in particular, miR-126-3p and miR-223-3p may be used for ischemic risk stratification after AMI. de los Reyes-García et al. [76] reported that miR-146 could be considered a promotor of thromboinflammation and recurrence in young patients with AMI. Chen et al. [47] reported that the expression levels of miR-4329 and miR-6718 were significantly lower in patients with myocardial infarction compared with controls. Based on these data, the authors concluded that these miRNAs could be used as potential biomarkers for AMI. Zhang et al. [80] demonstrated that miR-21 and MiR-208b promote cardiac fibrosis in AMI patients, activating the TGF- β1/Smad-3 signaling pathway. Wang et al. [44] investigated the role of miR-22 and miR-499 as sensitive biomarkers in the diagnosis of MI, confirming that these miRNAs could play an important role in early diagnosis. Wang et al. [77] investigated the role of miR-29: it was expressed less in subjects after MI. These authors demonstrated that the inhibition of this miRNA promoted angiogenesis, reduced fibrosis, and alleviated MI damage. Finally, Moric-Janiszewska et al. [56] identified miR-1, miR-133a, and miR-133b as potential diagnostic biomarkers of arrhythmia in pediatric patients.

### 3.3. miRNA Dysregulation in Heart Tissue Samples

Based on the literature review data, in the last five years, only three studies investigated miRNA dysregulation in heart tissue samples. These studies may be considered important because they were performed on human samples obtained after autopsy or tissue biopsies.

Yan et al. [66] investigated, in heart tissue, the role of four miRNAs (miR-133a-3p, miR-223-3p, miR-499a-5p, miR-3113-5p) as potential biomarkers for the diagnosis of sudden cardiac death (SCD). Based on their results, all miRNAs were significantly upregulated in the SCD group, suggesting their use in the post-mortem investigation in cases of SCD.

In agreement with this study, Pinchi et al. [29] investigated the role of miR-1, miR-133, miR-208, and miR-499, concluding that all miRNAs were higher in subjects who died from SCD compared with control. Moreover, the same authors compared the SCD group with the acute myocardial infarction (AMI) group, concluding that the expression levels of miR-1, miR-208, and miR-499 could distinguish between SCD and AMI.

Santos et al. [39] analyzed the miRNA profile of heart biopsies from atrial fibrillation (AF) patients, reporting that miR-130b-3p, miR-208a-3p, and miR-338-5p were differentially expressed in AF tissue samples.

### 3.4. miRNA Dysregulation and Increased Risks of Hypertension

Hypertension is a major health problem worldwide, considering that it is an important risk factor for heart disease and stroke; early identification could be very useful in order to prevent severe consequences in patients.

Suzuki et al. [60] reported that levels of miR-126, miR-221, and miR-222 were lower in subjects suffering from high blood pressure, suggesting that these miRNAs could be good candidates for hypertension prediction.

Miao et al. [73] concluded that miR-20a-5p, miR-93-5p, and miR-17-5p could have a potential value for chronic thromboembolic pulmonary hypertension with right ventricular dysfunction and injury.

Eikelis et al. [68], in their study, investigated the role of miR-132, describing a negative correlation between cardiovascular and metabolic diseases. Particularly, the reduction of miR-132 levels could be considered a target for the regulation of liver lipid homeostasis, with the possibility to act on the control of obesity-related blood pressure.

Mihaleva et al. [55] investigated the role of six miRNAs, demonstrating that their levels are higher in the tested group compared to controls. In particular, the expression levels of miR-155-5p and miR-424-5p were significantly higher, suggesting their pivotal role as promising biomarkers for cardiovascular damage in patients with type 2 diabetes mellitus. Mayer et al. [28], in their experimental study, demonstrated that a low expression of circulating miR-19a reflected a substantial additional mortality risk in stable cardiovascular patients.

### 3.5. The Most Investigated miRNAs: The Role of miR-133a-3p, miR-21, miR-499a-5p, miR-1, and miR-126

As previously described, the most studied miRNA is miR-133a-3p. Based on a recent in vitro study, considering that cardiac hypertrophy was induced by Ang II, the expression of miR-133a-3p was repressed in Ang II-treated HCM cells; contrariwise, its overexpression may be a promising strategy for cardiac hypertrophy treatment [87]. Its role has been investigated as an MI biomarker, but it has been concluded that it is not more specific when compared with troponin [88]. Moreover, this miRNA has been investigated as a regulator of angiogenesis, obtaining conflicting results. Nevertheless, its role could be functional in the regulation of diseased endothelial cells, leading to new therapeutic interventions in the treatment of patients suffering from cardiovascular pathologies that occur with excessive or insufficient angiogenesis [89]. Interestingly, this miRNA has been identified as a predictor biomarker for familial hypercholesterolemia as a substrate of CVD, considering that its elevated plasma levels anticipate these values within the following 2 years (average). This miRNA acts directly through lipid and inflammatory signaling in key cells for atherosclerosis progression [90].

MiR-21 is an essential regulator for cardiac homeostasis, acting as a cell–cell messenger with diverse functions. Considering its pivotal role in cardiac function regulation, it has been frequently investigated, taking into account its prospects in clinical therapy. Particularly, it exhibits fundamental functions in the cardiovascular system by targeting different mRNAs [91]. MiR-21 is involved in various cardiomyopathies (such as heart failure, dilated cardiomyopathy, myocardial infarction, and diabetic cardiomyopathy). Its levels notably change in both heart tissue and blood circulation, providing cardiac protection after heart injury [92]. It is noteworthy that in various papers, this miRNA was found more expressed in the infarct zone in mouse heart tissue exposed to AMI; this miRNA is involved in myocardial fibrosis post-MI, promoting the transforming growth factor-beta 1 (TGF-β1)-induced fibroblast activation, with the subsequent increased expression of Collagen-1, alpha-smooth muscle actin (α-SMA), and F-actin; on the other hand, miR-21 attenuated fibrotic processes [93].

In 2015, miR-499a-5p was described as a novel and sensitive biomarker of AMI. Based on this study, this miRNA may be used as a useful marker for early diagnosis of AMI [94]. The same miRNA seems to be involved in the prevention of DOX cardiotoxicity: as recently described, it could directly target p21, attenuating DOX-induced mitochondrial fission and apoptosis [95]. Similar results were reported by Shi et al., as based on their results, this miRNA is expressed in cardiomyocytes, and its expression was increased after AMI. These authors concluded that it plays a pivotal role in cardiomyocyte injury induced by hypoxia/reoxygenation (H/R); however, its pathway remains unclear [96].

One of the first studies that ascertained the pivotal role of miR-1 on cardiac contractile function and the potential molecular mechanisms was published in 2012. This study provides the first evidence that this miRNA dysregulation can cause adverse structural remodeling of the heart [97]. Later, it was reported that MirR-1 expression may induce atrophy, imparting fine-tuning of gene expression, as a part of redox signaling that leads to phenotypic alterations on heart tissue [98]. This miRNA was expressed in a different manner, allowing the differentiation between hypertrophic cardiomyopathy (HCM) and dilated cardiomyopathy (DCM), considering that it was downregulated in HCM. Moreover, miR-1-3p levels are correlated with the left ventricular end-diastolic diameter (LVEDD) and left ventricular ejection fraction (LVEF), which reflect the cardiac function in HCM [99]. One of the first studies that ascertained the pivotal role of miR-1 on cardiac contractile function and the potential molecular mechanisms demonstrated that the knockdown of miR-1 could mitigate the adverse changes in cardiac function associated with the overexpression of miR-1.

The circulating levels of miR-126 could be indicative of CVD; indeed, in several studies, it has been described that reduced levels of this miRNA are found in patients with HF compared with healthy controls. For these reasons, high levels of miR-126 may be related to better clinical conditions of patients affected by CVD [100]. In the same way, other authors described that low serum levels of circulating miR-126 were associated with an increased risk of premature death, suggesting that changes in serum levels of circulating miRNAs could be related to risk factors of premature death events [100,101]. In agreement with these studies, other authors described that low levels of this mRNA are related to increased amounts of inflammatory factors that could mediate the insurgence of CAD and atherosclerosis. These characteristics may suggest that this miRNA could be a future therapeutic target to reduce endothelial inflammation, thus decreasing the chances of developing CAD [102].

## 4. Conclusions

To date, CVD is one of the most important causes of mortality worldwide, thus the need for effective preventive strategies is imperative. Moreover, especially in developed countries, given that the average age of life tends to be higher, and considering that aging is associated with significant changes both in cardiovascular structure and function, the identification of new molecular markers remains one of the most important fields to be studied [103,104,105]. Particularly, the identification of markers capable of being sensitive in order to identify clinical signs and symptoms early to reduce morbidity and mortality from CVD remains a challenging research topic.

Analyzing the results of this review, most papers focused on miRNAs as promising therapeutic targets and biomarkers of drug responses and/or therapeutic approaches. The majority of the selected studies focused on circulating miRNAs, analyzing their expression levels in serum or plasma samples.

Only three studies were performed evaluating miRNA expression in human cardiac tissue after myocardial infarction. Two studies were performed in post-mortem samples: the investigation performed in post-mortem samples is an important research field considering the strengths of using autoptic tissues [29,66]. As demonstrated in other contexts [106,107], thanks to post-mortem findings, it is possible to perform an experimental investigation in tissues sampled from subjects who had died with an exact cause of death. In our opinion, the evaluation of expression values of the selected miRNAs could be carried out considering that it is performed on tissue lesions and that miRNAs are stable in FFPE samples [108]. For example, if the goal of the experimental study is monitoring heart tissue after MI, during autopsy, it is possible to select the exact area affected by infarction. In this way, it is possible to evaluate the expression values of the miRNAs by comparing the values in damaged tissue with normal tissue without the variability among subjects. We believe that in the near future, the use of post-mortem samples could be important to identify promising biomarkers of organ damage [109]. Moreover, thanks to the opportunity to analyze FFPE tissue, it could be possible to use post-mortem samples collected for other purposes, such as histological investigation to establish organ damage. In this way, forensic institutes are an invaluable “magic box” to obtain samples, underlying the importance of respect for ethical issues, such as the Declaration of Helsinki. Notably, the concept of a Biobank is not completely new: to date, population-based biobanks have been used to obtain extensive phenotypic and genotypic data; nevertheless, to date, the miRNA tools on biological samples stored in biobanks are still poorly investigated. Moreover, usually, only a few biobanks are based on tissue samples, usually stored blood samples. Furthermore, the biobanks that store tissue samples work predominantly in the cancer research field. Finally, it is important to note that several developed countries have not instituted national tissue biobanks [110,111,112].

The international scientific community has worked hard to elucidate the mechanisms by which miRNAs exert regulatory effects on gene expression, regulating physiological and pathological mechanisms. While it is clear that miRNAs are powerful gene regulators, all the underlying mechanisms remain unclear. The need for up-to-date data always justifies the enormous amount of scientific work in order to increasingly highlight their pathways. Given the importance of CVDs, miRNAs could be important both as diagnostic and therapeutic (theranostic) tools. In this context, the discovery of “TheranoMIRNAs” (new terms to describe the miRNAs that may be used both for diagnostic and therapeutic purposes) could be decisive in the near future. The definition of well set out studies is necessary in order to provide further evidence in this challenging field.

## Figures and Tables

**Table 1:** Main findings of the systematic review. The results are divided into columns: author, type of study, miRNAs involved, target (genetic, pathway, cellular), type of regulation (up/down), and clinical consequences. HCM—Hypertrophic cardiomyopathy, MI/R—myocardial ischemic reperfusion, ACS—acute coronary syndrome, MVD—multivessel disease, HF—Heart failure, AF—Atrial fibrillation, CAD—coronary artery disease, DCM—idiopathic dilated cardiomyopathy, CVE—Cardiovascular event, CVD—Cardiovascular disease, ACAS—Asymptomatic carotid artery stenosis, ARVC—Arrhythmogenic right ventricular cardiomyopathy, SCD—Sudden cardiac death, MI—Myocardial infarction, CTEPH—Chronic thromboembolic pulmonary hypertension, FGFR1—Fibroblast growth factor receptor 1.

First Author, Year, and Country	Study Model	miRNAs Investigated	Biological Sample/Target	Dysregulation (Up/Down)	Effect of Dysregulation
Bayés-Genis et al., 2018, Spain [23]	Observational prospective study	miR-22-3p, miR-133a-3p, miR-133b, miR-208a-3p, miR-320a, miR-345-5p, MiR-378a-3p, miR-423-5p, miR-499a-5p, MiR-622, miR-1254, MiR-1306-5p	Serum/CCAR1 (cell division and apoptosis regulator protein 1)	up (miR-1254, miR-1306-5p)	Significantly associated with increased in-hospital heart failure (HF) death.
Guo et al., 2018, China [24]	Case-control	miR-133a, miR-221	Plasma/Heart Failure with Reduced Ejection Fraction (HFrEF) gene	down	HF diagnostic biomarkers in elderly patients
Masson et al., 2018, Italy [25]	Randomized clinical trial	miR-132	Plasma/anti-hypertrophic transcription factor FoxO3	down	Prediction of HF severity
Li et al., 2019, China [26]	Case-control	miR-208, miR-494, miR-499, miR-1303	Plasma/PTEN, ROCK1	down	miR-208, miR-494, miR-499 and miR-1303 can be used as markers of myocardial infarction (MI), however, they do not have a higher value than traditional troponins
Liu et al., 2019, China [27]	Cohort	miR-150-3p, miR-197-5p, miR-320a, miR-494-3p, miR-939-5p, miR-1268a, miR-1275,	Plasma/Cardiomyocytes	up	Increase in HF adverse events
Mayer et al., 2019, Czech Republic [28]	Observational prospective study	miR-1, miR-19a, miR-21, miR-34a, miR-126, miR-133a, miR-197, miR-208b, miR-214, miR-223, miR-499	Serum/Inflammatory cells (increased fibrosis and apoptosis)	down (miR-19a)	In patients with carotid artery stenosis (CAS), a low level of miR-19a is an independent risk factor for mortality
Pinchi et al., 2019, Italy [29]	Case-control	miR-1-3p, miR-133a-3p, miR-208a-3p, miR-499a-5p,	Heart tissue/Ion channels	down (miR-1, miR-208) up (all in SCD)	Biomarkers of sudden cardiac death (SCD) due to early acute MI
Zhang et al., 2019, China [30]	Case-control	miR-155	Serum/Genes involved in pulmonary fibrosis and CAD (coronary artery disease).	down	Patients with HF after MI have elevated levels of these miRNAs
Zhu et al., 2019, China [31]	Retrospective cohort	miR-182-5p miR-5187-5p	Plasma/VEGF (vascular endothelial growth factor).	up	Diagnostic biomarkers for CAD
Asulin et al., 2020, Israel [32]	Case-control	miR-145-5p, miR-199a-5p, miR-5701	Human genes of development, cell growth, differentiation, proliferation, apoptosis, metabolism, and tissue remodeling	up (rheumatic valvulopathy) down (idiopathic valvulopathy)	Useful for differentiating the etiology of rheumatic from idiopathic valvulopathies
Barbalata et al., 2020, Romania [33]	Original article	miR-92a-3p miR-142-3p, miR-155-5p, miR-223-3p,	Plasma/TGF-beta2	(miR-92a) Down (miR-142, miR-155, miR-223) up	Prediction of CVD in patients with peripheral arterial disease
Ben-Zvi et al., 2020, Israel [34]	Case-control	miR-21-5p, miR-92b-3p, miR-125b-5p, miR- 133a-3p	Serum/cardiomyocytes	up (miR-125b-5p, miR-133-3p), down (miR-21-5p, miR-92b-3p)	Increased incidence of HF
Elbaz et al., 2020, France [35]	Case-control	miR-16, miR-92a, miR-122, miR-150, miR-186, miR-195, miR-223-5p,	Serum/Inflammatory cells (increased fibrosis and apoptosis)	up	Biomarker risk of ACS (acute coronary syndrome).
Ling et al., 2020, China [36]	Case-control	miR-21, miR-126	Serum/PTEN	(miR-21) down (miR-126) up	Biomarker of ACS
Liu et al., 2020, China [37]	Case-control	miR-1-3p, miR-20b-5p, miR-30b-5p, miR-142-3p, miR-1273g-3p, miR-6515-3p, miR-6793-5p, miR-7109-3p,	Serum/MAPK signaling pathway	up	Involvement in the pathogenesis of angina (stable and unstable)
Nie et al., 2020, China [38]	Case-control	miR-4281 miR-4763-3p	Plasma/KEGG related to apoptosis (TGF-β, mTOR, insulin, MAPK, p53)	up	Potential biomarker of fulminant myocarditis
Santos et al., 2020, Denmark [39]	Case-control	miR-130b-3p, miR-208a-3p, miR-338-5p	Heart biopsy/ion channel genes, extracellular matrix genes	down (ion channel genes) up (extracellular matrix genes)	Involvement in the development of AF
Silverman et al., 2020, USA [40]	Case-control	miR-29a-3p, miR-30a-5p, miR-150-5p	Plasma/Inflammatory cells (increased fibrosis and apoptosis)	up	Risk of increased SCD in patients with CAD
Su et al., 2020, China [41]	Case-control	miR-1	Serum/Endothelial function, angiogenesis and cell apoptosis	up	miR-1 within 3 h of acute chest pain is an independent risk factor for mortality in patients with MI
Turky et al., 2020, Egypt [42]	Observational prospective study	miR-133a	Plasma/FGFR1	up	Biomarker for early identification of stable CAD
Wakabayashi, 2020, Japan [43]	Case-control	miR-16-5p, miR-17-5p, miR-92a-3p miR-106a-5p, miR-135a-3p, miR-150-3p, miR-191-5p, mR-320b, miR-451a, miR-486-5p, miR-663b,	Serum/pro-inflammatory cytokines in foam cells and collagen synthesis in vascular smooth muscle cells	up	Increased incidence of ischemic heart disease
Wang et al., 2020, China [44]	Case-control	miR-22, miR-499	Serum/Cardiac myosin heavy chain gene	up	Sensitive and specific biomarkers for the diagnosis of MI
Weldy et al., 2020, USA [45]	Observational prospective study	miR-28- 3p, miR-371b-3p, miR-433-3p	Plasma/SMAD3 and 4, TGF-β1 and 2, E2F family transcription factors	up	Increasing right ventricular (RV) size and decreasing RV systolic function
Brundin et al., 2021, Sweden [46]	Case-control	miR-16-5p, miR-21-5p, miR-29-5p, miR-133a-3p, miR-191-5p, miR-320a, miR-423-5p	Serum/extracellular matrix proteins	up	Seven miRNAs were upregulated both in subjects suffering from idiopathic dilated cardiomyopathy (DCM) and ischemic heart disease (IHD)
Chen et al., 2021, China [47]	Case-control	miR-4329, miR-6718-5p	Plasma/MAPK, PI3K-Akt, Ras, Rap1 signaling pathway	down	Biomarkers for acute MI
Coban et al., 2021, Turkey [48]	Original article	miR-18a-3p, miR-130b-5p	Serum/SPP1 and TNFRSF11B genes	up	Biomarkers of CAD development
Elgebaly et al., 2021, USA [49]	Case-control	miR-106b miR-137,	Serum/Genes of Nourin	up	Biomarkers for early diagnosis of myocardial ischemia in patients suspected of CAD
Garcia-Elias et al., 2021, Spain [50]	Case-control	miR-22-5p, miR-199a-5p	Plasma/L-type Ca2+ channel, NCX and connexin-40	up	Decreased cardiac ejection fraction and increased incidence of AF
Gevaert et al., 2021, Belgium [51]	Observational prospective study	miR-181c	MAPK1, DNM2, and CDH1 (HFpEF pathophysiology)	up	Predicts response to exercise training in patients with HF
He et al., 2021, China [52]	Observational prospective study	miR-29b	Plasma/Inflammatory cells (increased fibrosis and apoptosis)	down	Independent risk factor for coronary artery calcification in patients with renal disease
Hromadka et al., 2021, Czech Republic [53]	Randomized clinical trial	miR-126-3p, miR-223-3p	VEGF, VCAM-1, SPRED1, PIK3R2/p85- beta, P2Y12, RPS6KB1/HIF1a	up	Independent risk stratification biomarkers for thrombotic events after MI
Lu et al., 2021, China [54]	Observational prospective study	miR-27b	Serum/Vascular smooth muscle cells	up	Prediction of the occurrence of ACS
Mihaleva et al., 2021, Bulgaria [55]	Case-control	miR-16-5p, miR-155-3p, miR-155-5p, miR-210, miR-221-3p, miR-424-5p	Serum/HIF1A (transcriptional regulator of the adaptive response to hypoxia)	up	Biomarker of cardiovascular complications in diabetic patients
Moric-Janiszewska et al., 2021, Poland [56]	Case-control	miR-1, miR-133a, miR-133b	Genes involved in the regulation of ion channels	up	Diagnostic biomarkers of arrhythmia in pediatric patient
Neiburga et al., 2021, China [57]	Original article	miR-10-5p, miR-10b-3p, miR-17-3p, miR-21-5p, miR-151a-5p, miR-181a-5p, miR-185-5p, miR-194-5p, miR-199a-3p, miR-199b-3p, miR-212-3p, miR-363-3p, miR-548d-5p, miR-744-5p, miR-3117-3p, miR-5683, miR-5701	Serum/AKT, PTEN and IRS1	down	Biomarkers of CVD
Sacchetto et al., 2021, Italy [58]	Case-control	miR-185-5p	Plasma/Inflammatory cells	up	Diagnostic biomarkers for ARVC (arrhythmogenic right ventricular cardiomyopathy)
Shen et al., 2021, China [59]	Case-control	Let-7b-3p, miR-21-3p, miR-28-3p, miR-99b-5p, miR-181c-3p, miR-133b, miR-320a, miR-500a-3p, miR-574-5p, miR-940, miR-1268b, miR-1307-3p, miR-4286, miR-4485-3p,	Serum/PI3K/AKT pathway	up (miR-4286)	Biomarker for increased risk of ACS
Suzuki, 2021, Japan [60]	Case-control	miR-126, miR-221, miR-222	Serum/NF-κB pathway	down	Increased incidence of hypertension
Szelenberger et al., 2021, Poland [61]	Case-control	miR-130b-3p, miR-142-3p, miR-146a-3p, miR-197-5p, miR-301a-3p, miR-338-3p, miR-3162-5p, miR-3656, miR-4299, miR-8069	Platelet/ARHGEF12, (regulation of actin cytoskeleton), AKT3 (focal adhesion), ARHGEF12 (vascular smooth muscle contraction)	5 miRNAs were upregulated (miR-301a-3p, miR-142-3p, miR-146a-3p, miR-130b-3p, miR-338-3p) and 5 miRNAs were downregulated (miR-8069, miR-4299, miR-3656, miR-197-5p, miR-3162-5p)	Potential platelet biomarker of ACS
Thottakara, 2021, Germany [62]	Case-control	miR-1, miR-495-3p, miR-499a-5p, miR-627-3p, miR-3144, miR-4454,	Plasma/Sarcomeric genes	up	Increased incidence of hypertrophic cardiomyopathy (HCM)
Tong et al., 2021, China [63]	Observational prospective study	miR-222	Serum/PI3K/AKT pathway	down	Increased incidence of MI/R
Xiao et al., 2021, China [64]	Case-control	miR-146a	Serum/S100A12	up	Biomarker for adverse prognosis of ST-Segment Elevation MI
Yamada et al., 2021, Japan [65]	Retrospective cohort	miR-21, miR-29a, miR-126	Serum/Inflammatory cells	up (miR-21 and miR-19a) down (miR-126)	Risk of premature death from cancer and CVD
Yan et al., 2021, China [66]	Case-control	miR-133a-3p, miR-223-3p, miR-499a-5p, miR-3113-5p,	Heart tissue/Inflammatory cells (increased fibrosis and apoptosis)	up	Sensitive biomarkers of SCD
Zhelankin et al., 2021, Russia [67]	Case-control	miR-21-5p, miR-17-5p, miR-146a-5p,	Plasma/cardiomyocytes	up (miR-21-5p, miR-146a-5p) down (miR-17-5p)	An increase in miR-146a-5p and miR-21-5p is an indicator of ACS, a decrease in miR-17-5p could be considered a general biomarker of CAD.
Eikelis et al., 2022, Australia [68]	Original article	miR-132	Serum/PTEN, SIRT1	down	Biomarker of hypertension in obese patients
Eyyupkoca et al., 2022, Turkey [69]	Case-control	miR-23b-3p, miR-26b-5p, miR-199a-5p, miR-301a-3p, miR-374a-5p, miR-423-5p, miR-483-5p, miR-652-3p	Plasma/Gene expression and remodeling of extracellular matrix	down (miR-301a-3p, miR-374a-5p) up (miR-423-5p)	Biomarker of adverse left ventricular remodeling after MI
Gager, 2022, Austria [70]	Observational prospective study	miR-125a (miR-125b, miR-223)	Plasma/Cardiomyocytes	up	Reduction of survival for ACS
James et al., 2022, Sweden [71]	Case-control	miR-224-5p	Extracellular Vesicles (EVs)/SMAD unit (TGF-beta pathway)	up	Biomarker of endothelial dysfunction in patients with low coronary flow reserve
J. Li et al., 2022, China [72]	Case-control	miR-203	Serum/Inflammatory cells (increased fibrosis and apoptosis)	up	Biomarker for early prediction of ST-Segment Elevation MI
Miao et al., 2022, China [73]	Retrospective study	miR-17-5p, miR-20a-5p, miR-93-5p, miR-665, miR-3202	Serum/Pulmonary artery smooth muscle cells and pulmonary artery endothelial cells (proliferation and apoptosis)	miR-20a-5p, miR-93-5p, miR-17-5p downregulated	Useful parameter in the diagnosis of chronic thromboembolic pulmonary hypertension (CTEPH)
Mompeón et al., 2022, Spain [74]	Observational prospective study	let-7g-5p, let-7e-5p, miR-26a-5p	Plasma/Involvement in the production of cytokines and chemokines	down	Potential biomarker of MI prognosis
Moscoso et al., 2022, Spain [75]	Observational prospective study	miR-125b, miR-499a,	Serum/KEGG related to apoptosis (TGF-β, mTOR, insulin, MAPK, p53)	up	Improvement of left ventricular ejection fraction after cardiac resynchronization therapy
de los Reyes-García et al., 2022, Spain [76]	Original article	miR-146a	Serum/TLR/NF-kB pathway	down	Contribution to thrombo-inflammation and MI recurrence in young patients
Wang et al., 2022, China [77]	Observational prospective study	miR-29	Serum/PI3K/mTOR/HIF1α/VEGF pathway	down	Development of MI
Yang et al., 2022, China [78]	Case-control	miR-29b (miR-let-7b)	Serum/Osteogenic transcription factors	down	Increased incidence of coronary artery calcification
Yu et al., 2022, China [79]	Case-control	miR-221, miR-222	Plasma/c-Raf/MEK/ERK pathway	up	Severity of ACS
Zhang et al., 2022, China [80]	Case-control	miR-21, miR-208b	Plasma/TGF-β1/Smad-3 Signaling Pathway	up	Cardiac fibrosis progression through activation of the TGF-β1/Smad-3 signaling pathway
Zhou et al., 2022, China [81]	Observational prospective study	miR-133a	Serum/FGFR1	up	Biomarker for early identification of stable CAD

## Data Availability

Data sharing not applicable as no new data were created or analyzed in this study.
